# Isoflurane for difficult sedation in critically ill children: a retrospective analysis in a mixed pediatric intensive care population

**DOI:** 10.3389/fped.2026.1810790

**Published:** 2026-05-28

**Authors:** Richard Biedermann, Melanie Koeplin, Claus Doerfel, Natja Liebers, Lars Newman, Hans Proquitté

**Affiliations:** 1 Department of Pediatrics, Division for Neonatology and Pediatric Intensive Care, University Hospital Jena, Friedrich Schiller University Jena, Jena, Germany; 2 Medical Faculty, Friedrich Schiller University Jena, Jena, Germany; 3 Department of Pediatrics, Division for Neuropediatrics, University Hospital Jena, Friedrich Schiller University Jena, Jena, Germany

**Keywords:** critical illness, delirium, isoflurane, sedation, withdrawal

## Abstract

**Introduction:**

When standard intravenous regimens fail, children in the pediatric intensive care unit often face escalating sedative polypharmacy with potential harm; we therefore evaluated inhaled isoflurane as a rescue sedation strategy in mechanically ventilated children with failure of a conventional sedation regimen.

**Methods:**

In this single-center retrospective observational study (2017–2020) in a 10-bed tertiary mixed pediatric intensive care unit (medical, neurologic, cardiac and traumatic conditions), isoflurane was delivered via an anesthetic conserving device while concomitant intravenous sedatives were down-titrated to sedation targets assessed by the COMFORT-B score, and cardiovascular support was quantified using the vasoactive-inotropic score.

**Results:**

Twenty-one children were analyzed (median age 3.0 years; median Pediatric Index of Mortality 3 score 15.9%); isoflurane was initiated after a median of 3 days of prior sedation and continued for a median of 8 days; the median number of sedative agents decreased from 4 to 3 (*p* < 0.001), doses of clonidine, sufentanil, and propofol were significantly reduced, and rescue medication use for breakthrough agitation declined from 100% to 42.9% (*p* < 0.001), while vasoactive-inotropic score increased from 6.22 to 9.70 (*p* = 0.008); five patients died, and among survivors delirium (87.5%) and withdrawal (44%) were frequent, with most extubated within 24 h after isoflurane cessation.

**Discussion:**

Isoflurane can meaningfully reduce sedative burden and agitation-related rescue interventions in difficult-to-sedate children, but the observed increase in vasoactive support and frequent delirium/withdrawal after prolonged use underscore the need for vigilant hemodynamic monitoring and structured down-titration—key considerations for centers contemplating volatile-based rescue sedation.

## 1 Introduction

Sedation management in critically ill children represents a complex clinical challenge in pediatric intensive care units (PICUs). While light sedation with preserved spontaneous ventilation is recommended for most ventilated patients, inadequate sedation can result in ventilator asynchrony or dangerous agitation of children (1). Treatment of critically ill patients with acute respiratory distress syndrome (ARDS) or traumatic brain injury (TBI) often requires deeper sedation (1–3). Prolonged sedation frequently leads to tolerance, necessitating increased medication doses and additional sedative agents (4, 5). Some medications commonly used in adult intensive care units, in particular propofol and ketamine, have dosing and duration limits in children, reducing medication options (6, 7). Midazolam, commonly used in PICUs, is associated with high rates of post-sedation delirium. Recent data suggest increased mortality associated with the use of high-dose midazolam infusions (8–11). Commonly used medication combinations of alpha-2 agonists combined with opioids are sufficient for many children. However, in more critically ill children and during prolonged sedation, this regime often is insufficient (1), particularly if neuromuscular blockade is indicated (12). Adjunctive sedatives such as phenobarbital or chloral hydrate, which are occasionally added when standard regimes prove insufficient, lead to an increase in drug interactions, side-effects and delirium, especially in the context of polypharmacy (11).

Isoflurane is a volatile sedative agent with extensive safety data in pediatric general anesthesia spanning over three decades (13–15). It has a low side effect profile and can be easily titrated to a desired sedation depth while maintaining spontaneous breathing (16). Commonly described side effects include cardiovascular instability and hypotension, as well as self-limiting neurologic abnormalities. A feared complication is malignant hyperthermia (17, 18). The introduction of anesthesia conserving devices (ACDs), such as the SedaConDa filter has enabled prolonged ICU sedation with volatile agents (19). There are limited data in adults demonstrating that this is feasible and safe and may serve as an alternative to propofol (20–22). However, pediatric data relating to isoflurane use remain scarce and primarily focus on those with congenital heart disease (23–26), with limited studies in mixed PICUs. At our hospital, isoflurane serves as a rescue sedation when conventional protocols prove insufficient. The primary objective of the study was to evaluate whether isoflurane could provide adequate rescue sedation while reducing concomitant sedative requirements in a mixed PICU population. Secondary objectives included characterizing the side effect profile and clinical outcomes in this heterogeneous pediatric population.

## 2 Patients and methods

### 2.1 Study design, settings and ethics

This retrospective observational study received approval from local ethics committee (2021-2311-Daten; 07/20/2021, “Isoflurane-Data”), all procedures were followed in accordance with the standards of the responsible committee on human experimentation and with the Helsinki Declaration of 1975. We reviewed charts of all patients who received isoflurane as a rescue sedation in the PICU at the University Hospital Jena between 01/2017 and 12/2020. Our PICU is a ten-bed mixed unit caring for pediatric patients with medical, neurologic and surgical conditions, ranging in age from 1 month to 18 years.

Demographic and clinical data was extracted from the electronic medical record systems, SAP and COPRA.

Given the retrospective and exploratory nature of this study, no formal primary endpoint was pre-specified. For the purpose of this analysis, the reduction in the number of concomitant intravenous sedative agents was designated the primary descriptive outcome, as it most directly reflects the intended clinical objective of isoflurane as rescue sedation. All remaining comparisons are considered secondary and hypothesis-generating. Results should be interpreted accordingly, and *p*-values do not constitute confirmatory evidence.

### 2.2 Indications and contraindications for rescue sedation

Isoflurane was considered for rescue sedation when conventional sedation failed and when no significant contraindications, including neuromuscular diseases, severe hemodynamic instability or family history of malignant hyperthermia, existed. Written informed consent was obtained from parents or legal guardians after explaining the experimental nature of treatment.

Failed conventional sedation was operationally defined as: inadequate sedation despite combination therapy with clonidine (2 µg/kg/h), sufentanil (1.5 µg/kg/h), and midazolam (0.15 mg/kg/h); requirement for repeated adjunct sedation due to breakthrough agitation (at clinicians' discretion); or poor ventilator-synchrony with failed weaning attempts. Primary sedatives included sufentanil and clonidine, with midazolam added once maximum doses were reached. Propofol was limited to 24-hour continuous infusions, with subsequent bolus-only administrations.

Rescue medication for breakthrough agitation was defined as any unscheduled administration of a sedative or analgesic agent in response to clinical signs of agitation documented by bedside nursing staff, including a COMFORT-B score exceeding the target range or acute behavioural disturbance. The decision to administer rescue medication was at the discretion of the treating physician. Agents used included sufentanil, esketamine and propofol boluses.

### 2.3 Isoflurane delivery and institutional protocol

Isoflurane delivery utilized SedoConDa-S Filter (Sedana Medical, Sweden) with 50 ml dead space. For children with tidal volumes exceeding 200 ml without ARDS, filters were positioned at the Y-Piece of respiratory tubing. In patients with lower tidal volumes or ventilation difficulties, inspiratory limb placement eliminated dead space but resulted in loss of scavenging function and increased isoflurane wastage. FlurAbsorb scavenging system (Sedana Medical, Sweden) prevented environmental contamination through ventilator exhaust ports. Anesthesia gas monitors (OR + Multigas-Monitoring, Masimo, USA) provided continuous gas concentration monitoring.

Ventilation was performed using Servo-U respirators (Maquet Critical Care AB, Sweden). Per protocol, sufentanil was continued at a dose of 0.5 µg/kg/h to ensure analgesia. If clonidine had been used for more than three days, it was continued at half dose and tapered over three days. Isoflurane was initially titrated to a calculated mean alveolar concentration (MAC) of 0.5. The dose could be adjusted if the desired sedation depth was not achieved. Adjunctive sedation was only used in cases of severe breakthrough agitation.

### 2.4 Outcome and safety measurements

All children were continuously monitored, including arterial blood pressure and continuous temperature monitoring. Creatine kinase, renal and liver function parameters were checked daily after initiating isoflurane sedation. Blood gases were sampled at least eight hourly.

Validated scoring systems quantified key parameters. Severity of illness at admission was assessed using the Pediatric Index of Mortality Score version 3 (PIM-3), a composite measure to predict PICU mortality (27). Cardiovascular support was quantified using Vasoactive-Inotropic Scores (VIS), thereby combining the weighted doses of all vasoactive medications (28).

Sedation depth was assessed using Comfort-B Scores, a six-item validated score correlating with sedation levels in critically ill children (29). Delirium was assessed using the Cornell Assessment of Pediatric Delirium (CAPD), a validated, nurse-administered observational screening tool with established sensitivity and specificity for delirium detection in critically ill children across all age groups and developmental stages (30).

For all dose comparisons, the “before isoflurane” time point was defined as the 6-hour period immediately preceding isoflurane initiation, using the mean recorded doses during this interval. The “during isoflurane” time point was defined as the 6-hour period following commencement of isoflurane administration.

Initiation or escalation of vasoactive agents was at the discretion of the treating intensivist, guided by age-appropriate mean arterial pressure thresholds.

### 2.5 Statistical analysis

Statistical analysis was performed using SPSS version 28 with non-parametric methods due to non-normal data distribution. Results are presented as medians with interquartile ranges (25th–75th percentile). Group comparisons were conducted using the Wilcoxon-signed-rank test and the Friedman test for continuous variables. Categorical data were analyzed with the McNemar test. *Post-hoc* analyses for multiple testing were performed using the Bonferroni correction. Two tailed *p*-values of less than 0.05, adjusted for multiple comparisons, were considered statistically significant. A *post-hoc* power analysis for the primary comparison (reduction in number of sedative agents) was performed using the normal approximation for the Wilcoxon signed-rank test, with effect size calculated as r = Z/√N. After manuscript completion, Claude.ai (Anthropic Sonnet 4 Model) was used to analyze spelling, grammar and syntax and to propose edits that conform to standard scientific English.

## 3 Results

### 3.1 Cohort

During the study period, 1,572 patients were admitted to our PICU; of these, 25 received isoflurane, with four subsequently excluded due to enrollment in a multicenter randomized trial with significant protocol variations, yielding 21 patients for analysis (1.3% of all admissions). A detailed description of the cohort can be found in Table 1. Overall survival was 76.2%; the five non-surviving patients had a median age of 5.0 years (range 0.1–14.3) with primary diagnoses of ARDS (*n* = 2), refractory status epilepticus due to mitochondrial epileptic encephalopathy (*n* = 1), bronchopulmonary dysplasia and extreme prematurity (*n* = 1), and hypoxic-ischemic encephalopathy following drowning (*n* = 1). Causes of death included pulmonary hemorrhage (*n* = 1), multi-organ failure following stem cell transplantation (*n* = 1), refractory ventilatory failure (*n* = 1), irreversible epileptic encephalopathy (*n* = 1), and cerebral herniation (*n* = 1). All deaths were attributed to progression of underlying disease.

**Table 1:** Cohort characteristics.

Parameter	Cohort (*n* = 21)
Age, median years (IQR)	3.0 (0.8–5.6)
Weight, median kg (IQR)	12.7 (9.1–16.0)
Male sex, % (*n*)	42.9 (9/21)
PIM-3 score, median % (IQR)	15.9 (2.85–24.2)
Pre-existing condition, % (*n*)	76.2 (16/21)
Length of stay (PICU), days (IQR)	18 (16–26)
Primary diagnosis category, % (*n*)	
Respiratory	71.4 (15/21)
Cardiac	9.5 (2/21)
Neurologic	9.5 (2/21)
Traumatic	9.5 (2/21)
Duration of sedation before isoflurane, median (IQR)	3 (1; 8)
Duration of ventilation before isoflurane; median (IQR)	2.5 (1; 7.25)
Number of baseline sedative agents	4 (4–5)
Baseline agents used, % (*n*)	
Opioids (Sufentanil, Remifentanil)	21 (100)
Alpha-2 Agonists (Clonidine, Dexmedetomidine)	17 (81)
Propofol	15 (71.4)
Benzodiazepines (Midazolam, Lorazepam)	12 (57.1)
Esketamine	9 (42.9)
Barbiturates	5 (23.8)
Neuroleptic agents (Levomepromazine, Haloperidol)	3 (14.3)
Mortality *n* (%)	5 (23.8)

### 3.2 Isoflurane administration characteristics

Isoflurane initiation occurred after a median of three days of conventional sedation. Due to the low average bodyweight of the cohort, the ACD filter was positioned in the inspiratory limb rather than at the Y-Piece of the ventilatory tubing in 90% of patients. This necessitated relatively high rates of isoflurane with a median of 4.5 ml/h (IQR 1.23–6 ml/h). Isoflurane was used for a median duration of 8 days (IQR 5–12.3 days). The complete isoflurane data-set use can be found in Table 2. We defined all percentage-based safety outcomes relative to the surviving cohort (*n* = 16) unless otherwise specified. Our detailed protocol can be found in the supplement.

**Table 2:** Parameters of isoflurane use.

Parameter	Value
Placement of filter, Y-Piece: Inspiratory limb	2:19
Mean dose of isoflurane, median ml/h (Q1; Q3)	4.5 (1.23; 6)
Mean MAC, median (Q1; Q3)	0.8 (0.57; 0.95)
Need for rescue medications during isoflurane treatment, % (*n*)	42.9 (9/21)
Duration of isoflurane treatment, median days (Q1; Q3)	8 (5; 12.3)
Within survivors (*n* = 16)	
Incidence of delirium, % (*n*)	87.5 (14/16)
CAPD-Score, median (Q1; Q3)	18 (15.75; 22.15)
Withdrawal, % (*n*)	44 (7/16)
Extubation within 1 day after cessation, % (*n*)	81.3 (13/16)
New neurologic deficit, % (*n*)	0 (0/16)

### 3.3 Sedative burden and rescue medications use

**Figure 1:**
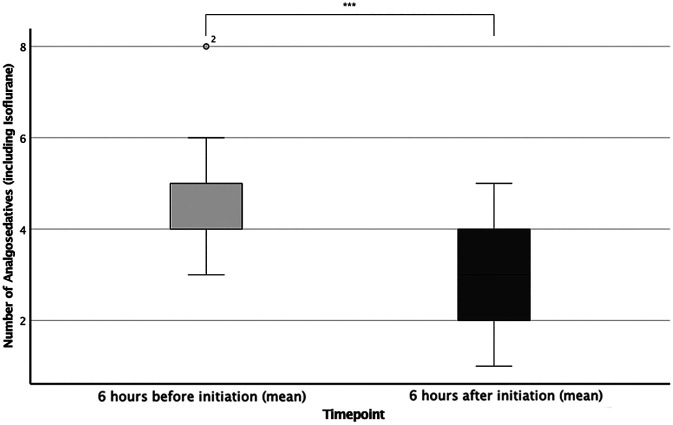
Comparison of sedative use in patients before and during the use of isoflurane administration. There was a significant reduction in the number of sedative agents per patient following the introduction of isoflurane as sedative agent. The median number of sedative agents decreased from 4 (4;5) before isoflurane to 3 (2;4) during its use with isoflurane included as one agent in the “during” timepoint *** (Wilcoxon Signed-Rank Test *p*-value < 0.001).

**Figure 2:**
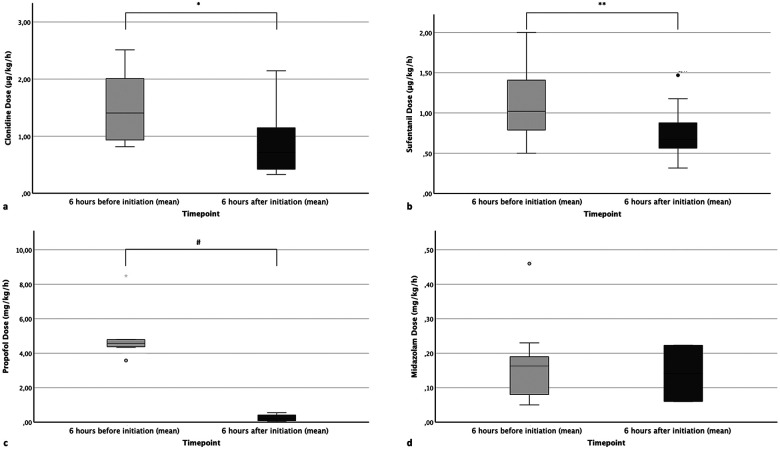
The doses of all analgosedatives could be reduced following commencement of isoflurane as a sedative, compared to the initial baseline. **(A)** Clonidine [1.40 (0.92; 2.20) vs. 0.72 (0.41; 1.17) µg/kg/h; *p* = 0.02]. **(B)** Sufentanil [1.02 (0.69; 1.45) vs. 0.67 (0.54; 0.91) µg/kg/h; *p* = 0.003]. **(C)** Propofol [4.46 (4.34; 4.8) vs. 0.008 (0.002; 0.09); *p* = 0.04]. **(D)** For Midazolam a trend in numerical dose reduction [0.163 (0.08; 0.21) vs. 0.14 (0.05; 0.27)] was shown. Statistical significance was determined using the Wilcoxon Signed-Rank test, with *p*-values indicated accordingly (* = 0.02; # = 0.04; ** = 0.003).

The introduction of isoflurane was associated with significant reductions in sedative medication requirements. The median number of sedative agents administered decreased from 4 (4;5) to 3 (2;4), (*p* < 0.001). To ensure a valid comparison, isoflurane was included as a sedative class in the “During Isoflurane” timepoint (Figure 1). Significant dose reductions were observed 6 h following commencement of isoflurane for clonidine (1.40 vs. 0.72 µg/kg/h; *p* = 0.02), sufentanil (1.02 vs. 0.67 µg/kg/h; *p* = 0.003), and propofol (4.46 vs. 0.008 mg/kg/h; *p* = 0.04). For Midazolam a non-significant reduction (0.163 vs. 0.14 mg/kg/h; *p* = 0.655) was observed (Figure 2). The most frequently co-administered drugs alongside isoflurane were sufentanil (85.7%) and clonidine (57.1%). The most frequently used medications for interventions were esketamine (66.7%) and propofol (38%). Rescue medication in response to breakthrough agitation was reduced from 100% (21/21) to 42.9% (9/21), (*p* < 0.001).

### 3.4 Sedation depth over time

Comfort-B scores remained stable during the initial 24 h of treatment. Baseline scores of 8 (7–9) decreased to 7 (6–8) at six hours post-initiation then increased to 10 (8–12) by 24 h (*p* = 0.368).

### 3.5 Hemodynamic effects and vasopressor requirements

**Figure 3:**
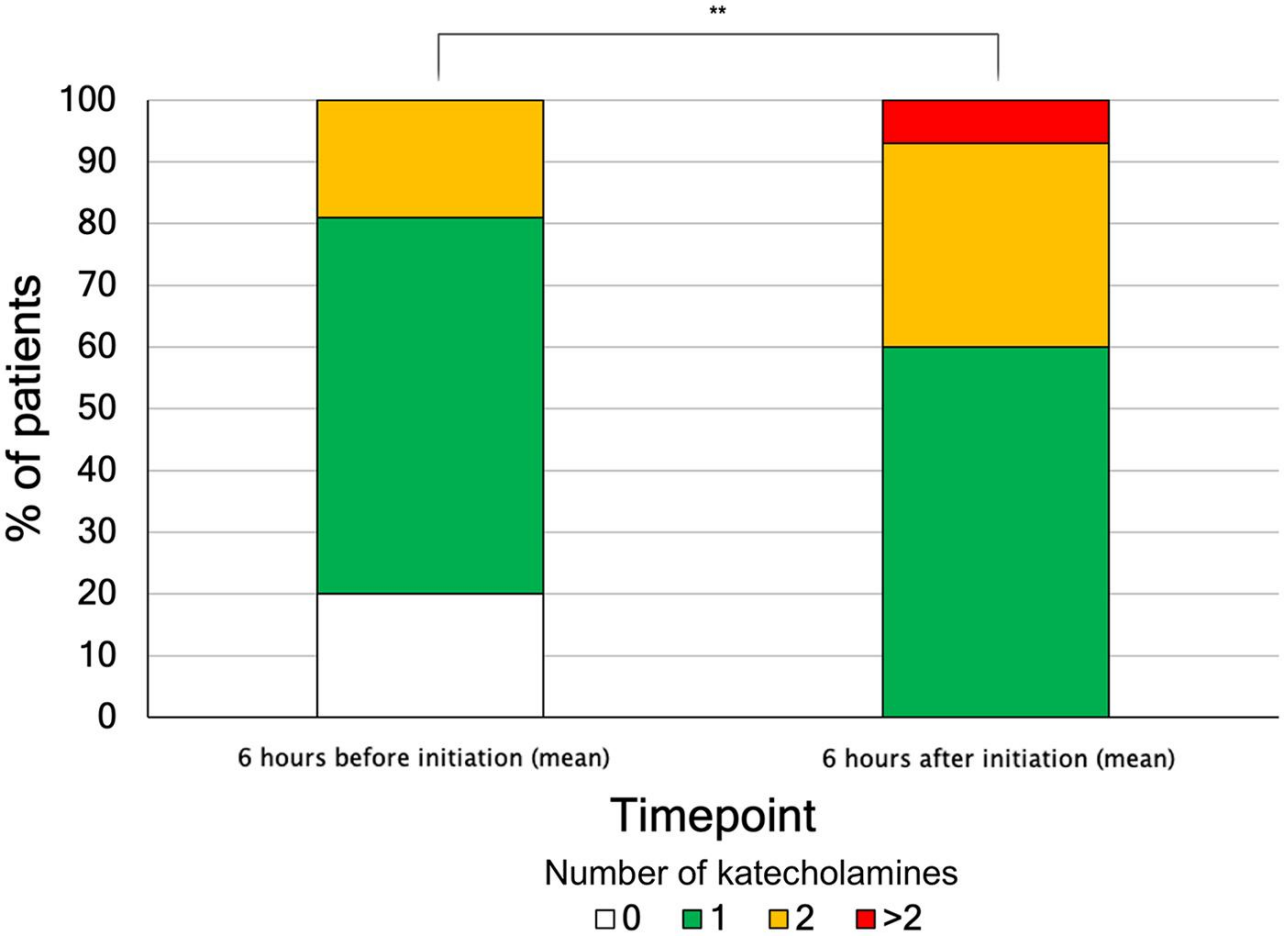
Increase in catecholaminergic agents following the introduction of isoflurane. The number of catecholamines used increased significantly during isoflurane treatment [1 (1;1) vs. 1 (1;2)]. ** Wilcoxon Signed-Rank Test *p*-value = 0.007.

**Figure 4:**
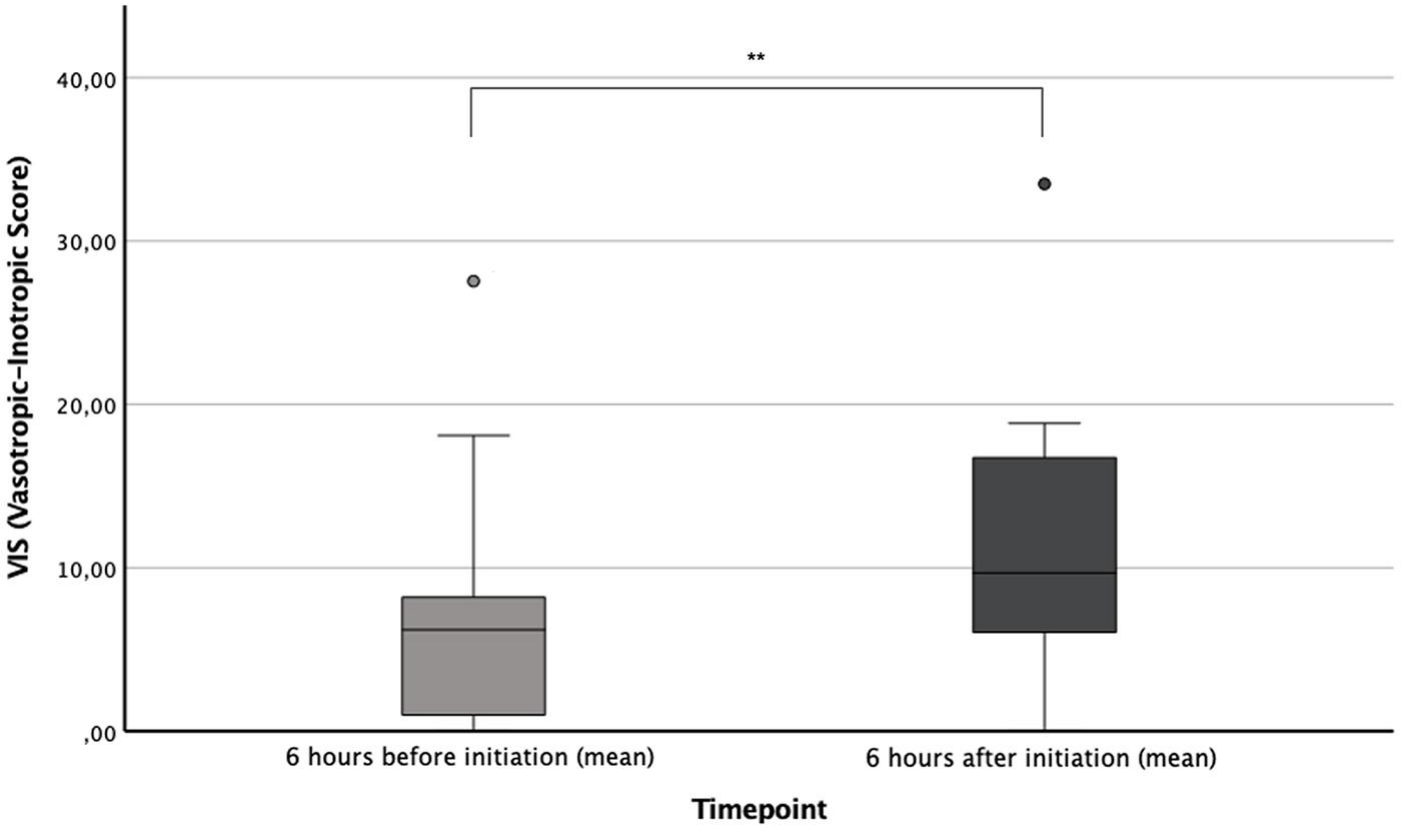
Vasoactive inotropic score (VIS) after introduction of isoflurane. There was a significant increase in VIS during isoflurane treatment [6.22 (0.75; 9.97) vs. 9.70 (5.43; 17)]. ** Wilcoxon Signed-Rank Test *p*-value = 0.008.

The cardiovascular impact of Isoflurane was demonstrated via significant increases in catecholamine demand. All patients required cardiovascular support following isoflurane initiation, with the median number of catecholamines increasing from 1 (1;1) to 1 (1;2) (*p* = 0.007). Correspondingly, VIS values increased significantly from 6.22 (0.75; 9.97) to 9.70 (5.43; 17) (*p* = 0.008) (Figures 3, 4). Following isoflurane initiation, 11 of 21 patients (52.4%) required either *de novo* vasopressor initiation (*n* = 4), a dose escalation of >50% in an existing agent (*n* = 4), or addition of an additional vasoactive agent (*n* = 3). The most frequently added agents were epinephrine (*n* = 5, 23.8%), norepinephrine (*n* = 4, 19.0%), and dobutamine (*n* = 4, 19.0%).

## 4 Discussion

This study provides one of the first reports on isoflurane as rescue sedation in a mixed pediatric intensive care unit treating patients with medical, neurologic, and surgical conditions. Our findings demonstrate three important outcomes: isoflurane use was associated with a significant reduction in concomitant sedative medications while achieving adequate sedation in all patients; commencement of isoflurane resulted in increased vasopressor support, demonstrating a cardiovascular impact; and unexpected withdrawal occurred in 44% of patients, contrasting with literature suggesting minimal withdrawal potential in short-term use. Of note, delirium rates were high (87.5%), which might reflect the complex patient population, rather than isoflurane-specific effects.

Sedation in the PICU remains challenging, with inadequate sedation leading to agitation, ventilator asynchrony, and adverse events, while many medications have dosing and duration limitations (1, 3, 4, 12). Rapid tolerance development frequently necessitates polypharmacy approaches and escalating doses leading to medication interactions, delirium as well as prolonged ventilation, and ICU stays (16). Our cohort included 21 patients who failed conventional sedation after a median of three days. All children (median age of 3 years) were critically ill, reflected by a median PIM-3 score of 15.9%. The median sedation duration with isoflurane was 8 days, representing genuine long-term sedation. Prior to isoflurane initiation, patients required a median of four intravenous analgosedatives - primarily sufentanil, clonidine, midazolam, and propofol or ketamine. Post-introduction, this was reduced primarily to sufentanil and clonidine, with sufentanil maintained at 0.5 µg/kg/h, recognizing isoflurane's limited analgesic properties (31). For patients on alpha-2 agonists for over 72 h prior to isoflurane therapy, a clonidine infusion was continued, with doses halved upon beginning isoflurane to mitigate withdrawal risks (32). Introduction of isoflurane was associated with significant dose reductions in clonidine (49%), sufentanil (34%), and propofol (99.8%). The significant reduction in sedative medication burden represents an important finding for pediatric critical care, particularly given the limited therapeutic options in children requiring prolonged sedation. This simplification addresses a key challenge by mitigating polypharmacy risks in PICUs. The ability to achieve adequate sedation while reducing breakthrough agitation medication requirements from 100% to 42.9% suggests clinical utility beyond simple medication substitution. Importantly, this experience in a mixed PICU population extends beyond previous pediatric studies that focused primarily on specialized cardiac populations, providing evidence for broader applicability across diverse pediatric conditions.

One of the main side effects of isoflurane is hypotension, primarily due to peripheral vasodilation and mild depression of cardiac function (17). The cardiovascular effects observed were more pronounced than typically reported in adult volatile sedation literature, with universal requirement for increased vasopressor support and significant VIS score increases (6.22 to 9.70; *p* = 0.008) (26, 33). The difference may reflect the high proportion of patients with systemic infections or post-cardiac arrest states (15 of 21), conditions causing distributive shock and thereby exacerbating the vasodilatory effects. Nevertheless, hemodynamic stability was maintained in all patients without therapy discontinuation, suggesting that cardiovascular effects remain manageable provided preparation and monitoring are appropriate. This finding is particularly relevant for mixed PICU populations where underlying pathophysiology may predispose to enhanced sensitivity to volatile agent effects.

The withdrawal symptoms observed in 44% of patients represent a novel finding that contrasts with literature describing minimal withdrawal potential with volatile agents (13, 25). Since we employed strict tapering protocols for all other medications these findings cannot be attributed to another medication class. This unexpected pattern required protocol modification to gradually reduce isoflurane doses over 24 h for patients receiving isoflurane for more than 72 h to minimize withdrawal and allow for gradual re-introduction of weaned medications. The high delirium rate (87.5%) with a median CAPD score of 18, well above the validated diagnostic threshold of 9, also exceeds that reported in adult studies, which demonstrate approximately 16% delirium incidence with prolonged inhalative sedation (34). Discerning whether this is attributable to isoflurane is difficult, as it likely reflects the cumulative effects of prior multi-drug sedation, prolonged exposure, young age, and severity of illness, rather than isoflurane-specific toxicity. Conventional sedation protocols had failed in all patients. Prior to isoflurane initiation four to six different agents were required, representing a fundamentally different population from adult volatile sedation studies or the recent IsoCOMFORT trial, which evaluated isoflurane as primary sedation rather than rescue therapy (20, 35). The emergence of these pediatric-specific phenomena highlights the importance of age-specific protocols and monitoring strategies. Despite these limitations, 81% of children could be successfully extubated within one day after cessation of isoflurane, even after a median use of eight days.

Several limitations affect the generalizability of our findings. The retrospective, single-center design introduces significant selection bias. The small sample size precludes robust statistical analysis, especially for rare events like malignant hyperthermia or neurologic complications. The absence of a control group prevents determination of whether reported outcomes represent improvement over an escalating intravenous sedation strategy. The heterogeneous patient population introduces multiple confounding variables that cannot be adequately controlled in an observational study of this size. Due to the small sample size and patient heterogeneity, no formal subgroup analyses by age group or primary diagnosis were performed. Cumulative MAC-hour exposure could not be calculated from the retrospective dataset, and no formal neurodevelopmental follow-up protocol exists for this cohort, precluding assessment of lasting effects of prolonged volatile agent exposure on the developing brain.

The clinical implications of these findings require careful consideration within the context of study limitations and the experimental nature of pediatric volatile sedation. Our experience suggests potential utility for isoflurane as rescue sedation in selected cases, not dissimilar to the IsoCOMFORT study, which showed non-inferiority to midazolam and no significant side effects in short term (<48 h) sedation (35). Implementation, however, should be considered only in tertiary centers with appropriate anesthesia expertise, comprehensive monitoring capabilities, and institutional protocols for volatile agent safety. The consistent requirement for cardiovascular support necessitates careful patient preparation, particularly in patients with underlying shock states or limited cardiovascular reserve. The withdrawal phenomena observed emphasizes the critical importance of systematic gradual weaning protocols for prolonged therapy. Healthcare teams must be prepared for enhanced delirium monitoring and management strategies, recognizing that former multiple sedative exposures may contribute to neurologic complications beyond isoflurane-specific effects.

Future research priorities should include prospective, multicenter studies with appropriate control groups to definitively assess efficacy and safety profiles in critically ill children. Investigation of optimal dosing strategies, systematic weaning protocols, and refined patient selection criteria would enhance clinical applicability. Economic analyses comparing volatile sedation costs and resource utilization with conventional approaches would provide important healthcare decision-making data. Future studies should incorporate comprehensive long-term neurodevelopmental assessments given ongoing concerns about anesthetic agent effects on developing nervous systems. This preliminary single-center experience contributes valuable initial safety and feasibility data to the extremely limited pediatric volatile sedation literature, representing one of the first reports of long-term isoflurane use in a mixed PICU population. Until more definitive evidence becomes available by well-designed prospective studies, isoflurane should be considered an investigational rescue option requiring careful individual risk-benefit assessment and comprehensive institutional protocol development. Because volatile-based sedation is used infrequently in many pediatric intensive care units and uncertainty regarding pediatric use persists, we provide our institutional standard operating procedure as Supplementary Material 1 as a practical implementation aid for centers with limited experience.

## Data Availability

The original contributions presented in the study are included in the article/Supplementary Material, further inquiries can be directed to the corresponding author.

## Supplementary material

Supplementary Data Sheet 1 Standard Operating Procedure Inhalative Sedation Jena.
