# Supplementary material for: Isoflurane for difficult sedation in critically ill children: a retrospective analysis in a mixed pediatric intensive care population
Source: Front Pediatr. 2026 May 28;14:1810790. doi: 10.3389/fped.2026.1810790 (PMC13253549; doi:10.3389/fped.2026.1810790)
Supplement: Supplementary Data Sheet 1 — Standard Operating Procedure Inhalative Sedation Jena. [file Datasheet1.docx]

**Protocol for the Use of Isoflurane in the PICU (University Hospital Jena)**

**Isoflurane may be considered in cases of failed conventional sedation when**:

1. Inadequate sedation despite combination therapy with clonidine (2 µg/kg/h*),* sufentanil (1.0 µg/kg/h*), and midazolam (0.15 mg/kg/h)
2. Need for repeated adjunct sedation due to breakthrough agitation
3. Poor ventilator synchrony and failed weaning attempts

**Informed consent:**

Obtain written informed consent from legal guardians.

**Check for contraindications, especially:**

- Personal or family history of malignant hyperthermia
- Adverse reactions during previous anesthesia
- Neuromuscular disorders (especially muscular dystrophy).

**Preparation:**

**Equipment Setup**

- Connect tubing, scavenging device, and anesthetic gas monitoring according to manufacturer guidelines.
- Filter Placement:
  - If desired tidal volume (4–8 ml/kg body weight) is < 200 ml or ventilation issues are present, insert the ACD filter in the inspiratory limb.
  - If not, the ACD filter can be placed at the Y-piece. Expect an increase in CO₂; adjust ventilation for the expected additional dead space (~50 ml for Sedaconda-S ACD filter) by increasing tidal volume or respiratory rate.

**Hemodynamic Preparation:**

- Ensure euvolemia and adequate cardiac function before initiating isoflurane.
- Arterial blood pressure monitoring is preferred; if unavailable, measure blood pressure every 3 minutes during induction.
- Prepare and connect norepinephrine infusion in case of hypotension.

**Initiation:**

- Flush tubing and filters per manufacturer instructions (e.g., 1.7 ml bolus for Sedaconda-S ACD filters).
- Begin with an isoflurane infusion of 1 ml/h; titrate by ±0.5–1 ml/h to achieve a calculated MAC of 0.5.
- Once MAC reaches 0.5:
  - Reduce sufentanil to 0.5 µg/kg/h (or 50% of the previous dose)
  - Reduce alpha-2 agonists to 50% of their previous dose.
  - Discontinue all adjunctive sedative regimens.
  - Alpha-2 agonists may be discontinued if they were administered for <72 hours.

**Titration:**

- Adjust isoflurane to reach the desired sedation level
- MAC is a calculated value; titrate actual dose to clinical sedation
- Literature generally accepts MAC values of approximately 0.3-0.5 (-1.0)
- Consider EEG monitoring to avoid burst-suppression, except when clinically warranted.

**Monitoring:**

- Assess sedation depth using Comfort-B every 30 minutes until stable, then every 4 hours.
- Blood gases every 8 hours.
- Daily monitoring: creatine kinase, liver, and renal function.
- In cases of impaired renal function: monitor fluoride levels daily.

**Termination of sedation:**

- Duration ≤48 hours:
  - Stop isoflurane infusion
  - Expect awakening within 30–60 minutes.
- Duration > 48hours:
  - Reduce isoflurane gradually over 24 hours
  - Adjust alpha-2 agonists to maintain sedation as needed.

**Post-extubation:**

- Monitor for withdrawal symptoms every 8 hours
- Evaluate for delirium every 8 hours
- Check for new neurologic deficits; consult pediatric neurology if present.

**Emergency Management: Malignant Hyperthermia**

**Warning Signs:**

- New-onset fever
- Muscle stiffness
- Unexplained increase in CO₂.

**If malignant hyperthermia is suspected:**

- Immediately disconnect the filter and stop isoflurane.
- Increase respiratory rate and set FiO₂ to 1.0.
- Administer dantrolene; call for help as administration is resource-intensive
- Obtain immediate laboratory tests and blood gases
- Correct volume status and acid-base disturbances.

* The lower sufentanil threshold in the current protocol (>1.0 µg/kg/h) compared to the dose used during the data collection period (>1.5 µg/kg/h) reflects a subsequent protocol revision following our observation of a high rate of gastrointestinal complications including ileus associated with prolonged high-dose opioid infusions.
